# Bicycle Data-Driven Application Framework: A Dutch Case Study on Machine Learning-Based Bicycle Delay Estimation at Signalized Intersections Using Nationwide Sparse GPS Data

**DOI:** 10.3390/s23249664

**Published:** 2023-12-07

**Authors:** Yufei Yuan, Kaiyi Wang, Dorine Duives, Serge Hoogendoorn, Sascha Hoogendoorn-Lanser, Rick Lindeman

**Affiliations:** 1 Faculty of Civil Engineering and Geosciences, Delft University of Technology, Stevinweg 1, 2628 CN Delft, The Netherlands; d.c.duives@tudelft.nl (D.D.); s.p.hoogendoorn@tudelft.nl (S.H.); 2 Faculty of Science, Mathematics and Computer Science, Universiteit van Amsterdam, 1090 GE Amsterdam, The Netherlands; 3 Mobility Innovation Center Delft, Delft University of Technology, Stevinweg 1, 2628 CN Delft, The Netherlands; s.hoogendoorn-lanser@tudelft.nl; 4 Rijkswaterstaat, Griffioenlaan 2, 3526 LA Utrecht, The Netherlands; rick.lindeman@rws.nl

**Keywords:** data-driven bicycle applications, GPS cycling data, machine learning, bicycle delays, signalized intersections

## Abstract

Data-driven approaches are helpful for quantitative justification and performance evaluation. The Netherlands has made notable strides in establishing a national protocol for bicycle traffic counting and collecting GPS cycling data through initiatives such as the Talking Bikes program. This article addresses the need for a generic framework to harness cycling data and extract relevant insights. Specifically, it focuses on the application of estimating average bicycle delays at signalized intersections, as this is an essential variable in assessing the performance of the transportation system. This study evaluates machine learning (ML)-based approaches using GPS cycling data. The dataset provides comprehensive yet incomplete information regarding one million bicycle rides annually across The Netherlands. These ML models, including random forest, k-nearest neighbor, support vector regression, extreme gradient boosting, and neural networks, are developed to estimate bicycle delays. The study demonstrates the feasibility of estimating bicycle delays using sparse GPS cycling data combined with publicly accessible information, such as weather information and intersection complexity, leveraging the burden of understanding local traffic conditions. It emphasizes the potential of data-driven approaches to inform traffic management, bicycle policy, and infrastructure development.

## 1. Introduction

In recent years, the bicycle has gained significant momentum in terms of everyday use and thus policy importance, with the Netherlands, Denmark, and Germany in the lead in terms of the highest bicycle usage [1,2]. This surge in popularity is partly due to the advent of e-bikes and the lifestyle changes brought about by the COVID era, leading to an unprecedented increase in cycling [3,4]. From a policy perspective, bicycles are increasingly considered a sustainable substitute for short- to medium-distance car journeys (up to 10 km for regular bikes up to 20 km for electric bikes) [5]. A growing number of cities (such as New York [6], Paris [7], Oslo [8], and Barcelona [9]) are prohibiting car traffic from their city centers to lower CO_2_ emissions and create more space for pedestrians and cyclists. Alongside public transport, bicycles offer a sustainable and efficient means of travel from one point to another. They also serve as a crucial link for first/last mile connectivity to public transport [5]. To encourage cycling, authorities—including public transport operators, provinces, and local municipalities—invest in high-quality cycling infrastructure, provide bicycle parking facilities at public transport stations, and launch cycling promotion campaigns. These concerted efforts aim to boost bicycle usage at a national level, as seen, for example, in the Netherlands.

As the construction of high-quality cycling infrastructure necessitates substantial investments, there is a corresponding rise in the demand for quantitative justification and performance evaluation of interventions in the cycling infrastructure. In recent years, the Netherlands has made significant strides in data-driven bicycle policy initiatives [2,10]. Among other achievements, this has resulted in establishing a national protocol for bicycle traffic counting. This protocol outlines procedures for conducting bicycle traffic counts and standardized methods for centrally storing this data at the Dutch National Data Warehouse [11].

Another valuable data source to support our understanding of cycling behavior is (revealed) travel data, including national survey data (such as the Netherlands Mobility Panel—MPN [12], which studies trends in the travel behavior of a fixed group of individuals and households over a long period (since 2013); the Travel Survey in the Netherlands—OViN [13]—which provides adequate information about the daily mobility of the Dutch population, according to place of origin and destination, time at which the transport takes place, means of transport used and travel motives for the journey (from 2010 to 2017)) and GPS cycling data. In the past, several initiatives have been launched to collect and process GPS bicycle data. Notable examples include the National Bike Counting Week (National initiative on cycling data collection using a smartphone application—FietsTelWeek, from 2015 to the present time) [14], RingRing (a cycling data collection app—allowing users to record and review their trips, and to be notified about upcoming traffic lights) [15], and Go Velo (a cycling stimulation program) [16].

The Talking Bikes program has been operational since 2020 [17], collecting GPS data. This program aims to connect bicycles to the surrounding infrastructure, for instance, intelligent traffic control installations (roadside traffic controllers) via the user’s mobile phone. The outcome of the Talking Bikes program is a comprehensive dataset of GPS cycling data, with over one million bicycle rides per year geographically distributed across the Netherlands.

However, merely collecting GPS data is insufficient to bolster cycling policy and traffic management. This data must be transformed into knowledge and actionable insights. The challenge lies in determining what specific knowledge and insights about contemporary cycling behavior and cyclists’ choices can be extracted from this GPS data. When examining the ecosystem surrounding cycling data, it is clear that various stakeholders—including governments, private entities, knowledge institutions, and platforms—hold divergent perspectives and interests. A prime example of such an ecosystem in the Netherlands is the collaborative initiative known as Tour-de-Force [18], which has an ambitious goal: an increase of 20 percent in the number of bicycle kilometers in 2027. Within this ecosystem, the exploration of cycling data, including counting or GPS data, begins with the data itself, and the focus is on how valuable insights can be derived from it. Conversely, from the policy-related demand side, examining the conditions that should be set for future data collection is necessary. However, there is a conspicuous absence of a generic framework or guideline on how cycling data can be harnessed to support traffic management and policy-relevant applications and extract relevant insights.

As one of these application examples, accurate determination of average delays and stops of road users, including vehicles, bikes, and pedestrians, is crucial for effectively managing a signalized intersection [19] and for adequate estimation of route choice models [20]. While field observation studies can determine this delay information, such an approach is costly and time-consuming. Analytical methods commonly used to estimate delay often fail to generate accurate estimates, particularly in conditions of oversaturated traffic flow [19]. Additionally, these traditional analytical methods would require site-specific traffic control factors/information (e.g., cycle durations, green/red times) and traffic demand factors (demand of local traffic users). This data is not generally available in a national database or is inaccessible to the public.

In recent years, delay estimation models based on machine learning (ML) have been introduced in the literature to estimate delays more accurately. However, these applications have primarily focused on vehicles [19,21], with limited extension to bicycle traffic in the existing literature. Moreover, the feed data for delay estimation models range from camera readings (image processing or manual counting) to GPS data (either from smartphones (the GSM network, or apps) or from on-bike trackers). The former data source might provide the ground truth information, but the data extraction process is time-consuming; the latter might only offer a subset of the total traveler population [22,23].

This article aims to bridge the research gap by presenting a generic data-driven framework for traffic management and cycling policy-relevant applications. Within this framework, we will demonstrate how cycling data can be utilized to gain strategic insights at a national level via a case study. In essence, we are developing intelligence to transform raw GPS tracks from individual bicycle sensors into policy support and decision-making tools. To achieve this, we estimate an ML-based model that can identify the average bicycle delay at signalized intersections using the relatively sparse GPS cycling data at a national scale from the Talking Bikes program, which is also considered a state-of-the-art approach. In this work, we develop estimation models ranging from simple linear regression to sophisticated ML approaches. These include random forest (RF), k-nearest neighbor (kNN), support vector regression (SVR), extreme gradient boosting (XGBoost), and neural networks (NN). These methods have not previously been applied in the literature for estimating bicycle delays at signalized intersections. Notably, we primarily consider this application using sparse cycling GPS data as an input, excluding local control and/or demand information for bicycles and other traffic users at intersections.

The remainder of this paper is organized into five sections. Section 2 presents a framework for data-driven applications for traffic management and policymaking. Section 3 elaborates on one of the specific applications of bicycle delay estimation, including the conceptual framework and model approaches of an ML-based case study. This is followed by Section 4, which describes the case setups for estimating bicycle delays at signalized intersections on a national scale. Section 5 presents the results and discussion, reflecting on how the case study can validate the proposed concept. Finally, Section 6 concludes the paper with a summary of the main findings and recommendations.

## 2. Framework for Bicycle Data-Driven Applications

We aim to explore the potential cycling data offers to traffic management and policy-relevant applications. In this context, we can identify five types of data-driven applications: (a) real-time traffic information and control (such as information provision, route guidance, speed limits); (b) planning and design for integrated multi-modal traffic management, which lays down the strategic basis (e.g., determining the functions of roads and the priority of modes and roads) for real-time traffic management (GebiedsGericht Benutten (in Dutch): GGB+) [24]; (c) transportation planning (e.g., demand estimation and prediction of transport services (first and last mile)); (d) network design (considering the design of bicycle networks, bicycle parking facilities, traffic management measures, and control plans); and (e) policymaking and assessment (ex ante and ex post policy assessment).

For each of these applications, we can utilize traffic and transportation data to calculate traffic variables, real-time inputs, or key performance indicators. To further consider various application fields, we broadly define six categories: *accessibility*, *reliability*, *safety*, *health*, *environment*, and *equity*. This results in specific combinations regarding the five applications and the six categories in a large matrix. The detailed combinations are generated according to the authors’ experience and inspired by existing studies in the literature. Detailed reasoning and support references on specific combinations of traffic variables and their applications can be found in the authors’ technical report [25]. In conclusion, the complexity and multi-faceted nature of cycling policymaking and traffic management necessitate diverse considerations. These considerations vary significantly across different applications, highlighting the need for a comprehensive and adaptable approach.

Note that for all the identified application combinations, we can further determine the extent to which data quality (such as *accuracy*, *reliability*, *sampling rate*, *aggregation level*, *latency*, and *privacy*, as a third dimension) is sufficient for a specific application. This three-dimensional structure will help set up guidelines and requirements for data collection and utilization for data/service providers and governmental parties.

For instance, when aiming to improve bicycle accessibility in specific regions or urban areas, from the GGB+ and network design perspectives, stakeholders might be interested in knowing bicycle delays and their distribution at signalized intersections. Given the current GPS data from the Talking Bikes program, by investigating its data quality (e.g., accuracy, sampling, and aggregation levels), we will demonstrate how this data can be utilized to estimate bicycle delays at intersections and gain strategic insights at a national level. It should be emphasized here that our primary objective is not to create the most precise estimation model for real-time monitoring of bicycle delays on a per-movement basis at signalized intersections but to make the best use of the existing data sources, taking into account their quality and capabilities, to extract potential insights for traffic management and infrastructure design. The next chapter will justify the focus of the paper.

## 3. Application on Bike Delay Estimation

This section first discusses the related work on bicycle delay estimation. Next, a generic conceptual framework that identifies possible influential factors on bicycle delays at signalized intersections is proposed based on evidence in the literature. Finally, the estimation approaches will be briefly described.

### 3.1. Related Work on Bicycle Delay Estimation

**Figure 1:**
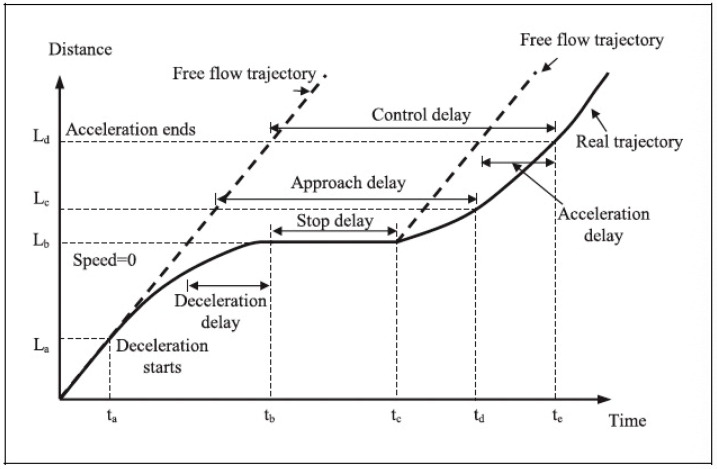
Demonstration of vehicle delays at a signalized intersection. This delay process for vehicles can be analogized to cycling. Source: [19].

Many studies have investigated vehicle delays at signalized intersections [19,21]. The actual delay experienced by vehicles at signalized intersections consists of three types: control delay, stop delay, and approach delay, as shown in Figure 1. This definition and concept are widely used to describe vehicle delays, and we believe a similar process is applied to bicycle traffic. Only a few studies have focused (either entirely or partially) on cycling delays at signalized intersections. These works mainly rely on analytical approaches to infer the observed delays through bicycle trajectories.

Velthuijsen (2020) directly calculated and estimated bike delays from smartphone GPS data in a Dutch city based on fundamental laws of physics [26]. This study considers three scenarios of defining reference speed values (as the ‘free-flow’ speed). Similarly, Rupi et al. (2020) and Poliziani et al. (2022) inferred bicycle waiting times (including delays) based on a predefined threshold speed, using relatively rich GPS traces at a city scale in Italy [20,27]. The novelty of these studies lies in a preprocessed map-matching operation. Gillis et al. (2020) measured bike delays at signalized intersections by interpolation between GPS locations before and after the intersections [5], relying on data from the Bike Count Week in Belgium [14]. A similar approach to deriving delay times can be found in studies in Canada [28] and Sweden [29]. No dedicated estimation models were developed in these studies to capture and reproduce generic delay behavior.

Yuan et al. (2019) developed a linear regression model to estimate the start-up lost time as part of approach delay (which is used to calculate bicycle intersection capacity) from empirical trajectory data derived from cameras at a specific intersection in Amsterdam [30]. Few attempts have been made to utilize ML models for bicycle delay estimation and inference. Reggiani et al. (2020) successfully employed an NN model to estimate individual cyclist travel times, considering various scenarios, including approach delay, stop delay, and/or control delay [31]. Their study utilized data from cameras, loop detectors, and control signals in Utrecht. Although ML has demonstrated significant potential in enhancing traffic state estimation, its application for bicycle delay estimation remains limited, especially in scenarios involving sparse GPS data at a large (national) scale.

### 3.2. A Conceptual Framework for Determining Bike Delays at Signalized Intersections

**Figure 2:**
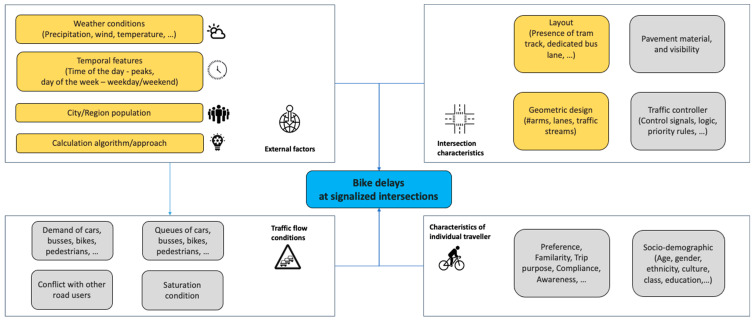
Conceptual framework for bike delays at signalized intersections. The highlighted influential variables are included as the independent variables in the case study.

We proposed a conceptual framework of all the most relevant factors to explain bicycle delays at intersections, as shown in Figure 2. We have identified four categories: characteristics of individual travelers, intersection characteristics, traffic flow conditions, and external factors.

The characteristics of individual travelers play a considerable role in determining traffic variables, such as travel time and delay. The traveler heterogeneity in terms of desired speed, movement direction, interaction preference, and maneuver flexibility is more considerable for bicycle traffic than car traffic in most situations [20]. For instance, in most cases, it is challenging to accurately determine whether a cyclist is following another cyclist and being constrained by their speed. This uncertainty extends to not knowing if cyclists travel at their desired speed or slower speeds. Even if we could identify a cyclist following another, we lack knowledge about their desired speed, so the actual delay is never directly observable [32].

The characteristics of intersections are identified as another important attribute. The geometry of the design, layout, pavement, and visibility will influence the extent to which the cyclist interacts with other road users as well as with the environment [20,28,29]. The controller scheme (e.g., signals, logic, priority rules) contributes the most to the stop delay and control delay at signalized intersections [5,30,31]. Additionally, the local traffic conditions need to be considered, such as the demand and queue length of various transport modes, the state of being saturated or oversaturated, because the control signals may assign more green times to the mode with greater priorities.

The influencing factors on bicycle delays also include some variables that are not governed by the local conditions (regarding travelers, intersections, and traffic flow), such as climate [33,34], temporal features [20,34,35], and demographic features (population) [36]. Weather can be considered one of the most significant external factors in cycling trip generation, comfort, and efficiency [28,33,34]. Additionally, various approaches and algorithms play a role in computing the magnitude of delays [5,20,28,29].

### 3.3. Intersection Delay Estimation Approaches

The GPS data collected from the Talking Bikes program provides valuable insights into annual nationwide bicycle rides. However, these recorded rides may not always offer a comprehensive representation due to geographical coverage and penetration rate limitations. Consequently, the data may not capture the full spatiotemporal range of a specific intersection, which hinders the application of an analytical approach to accurate estimation of delays. Considering the potential of machine learning to improve traffic state estimation, we propose an ML-based study that specifically targets the identification of average bicycle delays at signalized intersections using relatively sparse GPS cycling data nationwide. In this study, the estimation models include six variants, which are briefly described hereafter. Note that the five chosen ML models have indicated their validity for vehicle delay estimation to capture temporal trends and physical processes [18,20]; however, they have not previously been applied in the literature for bicycle delay estimation.

**Linear Regression (LR):** Linear regression is used to predict a dependent variable based on the values of one or more independent variables [37]. Linear regression is simple, fast, and often a good first algorithm for predictive modeling. In the current context, linear regression could predict bicycle delays based on variables such as time of day, weather conditions, and traffic volume.

**Random Forest (RF):** Random forest is a versatile ML method capable of performing both regression and classification tasks [38]. It is an ensemble learning method where a group of weak models combine to form a robust model. In random forest, multiple decision trees are created and combined to obtain a more accurate and stable prediction. It is highly flexible and can accommodate various data types. In our project, an RF model could be used to predict bicycle delays based on multiple features, with the advantage of being robust to outliers and non-linear data.

**K-Nearest Neighbors (kNN):** k-nearest neighbors is a simple, instance-based learning algorithm, used for classification and regression [39]. It determines the output of a new instance based on the majority vote or average of its ‘k’ most similar instances from the training dataset. The similarity is typically calculated using distance metrics such as Euclidean or Manhattan distance. For our project, kNN could be used to estimate bicycle delays by finding the ‘k’ of the most similar historical instances based on features such as time of day, weather, and traffic conditions.

**Support Vector Regression (SVR):** Support vector regression is a type of support vector machine that supports linear and non-linear regression [40]. SVR performs linear regression in a higher dimensional space, where the input data are mapped via a kernel function. It tries to find a line so that errors do not exceed the threshold. In bicycle delay estimation, SVR could be used to model complex, non-linear relationships between various factors and the delay times.

**Extreme Gradient Boosting (XGBoost):** XGBoost is a powerful ML algorithm for regression and classification problems [41]. It implements gradient boosting machines, which are ensemble models that aim to minimize prediction error by combining the predictions of multiple simpler models, called ‘weak learners’. XGBoost is known for its speed and performance. In our project, XGBoost could be used to predict bicycle delays by learning from previous delay instances and iteratively improving its predictions.

**Neural Networks (NN):** Neural networks are a set of algorithms modeled after the human brain, designed to recognize patterns [42]. They interpret sensory data through machine perception, labeling, or clustering of raw input. They can be trained to recognize complex patterns, make predictions, and make decisions in a way that is similar to human decision-making. Neural networks are particularly well-suited to problems where the inputs correlate highly with the target output. In our project, an NN could be trained to recognize complex patterns in the data and predict bicycle delays based on various input features.

## 4. Introduction to the Delay Estimation Case Study

This section describes the case study, including the definition of both dependent and independent variables, the configuration of the estimation models, and the assessment criteria.

### 4.1. Dataset

GPS cycling data generally registers changes in locations and time instants, with no distinction being made based on the mode of transport or motive of individuals. The trip samples might come from cyclists who use bike apps (Ring-Ring), shared bike users, or company bike users (via bike trackers—Tracefy). It contains approximately 3 million bicycle rides across the country over two years. While the overall dataset size is substantial, it is worth noting that the distribution of trips at specific intersections over two years may be limited. The average number of daily records is around 4000, distributed across the country. The sampling rate varies per trip and data record, starting from 1 second (s) to 30. This justifies the ‘sparse’ feature of this GPS dataset.

For demonstration purposes, we selected 18 intersections, namely three intersections from six Dutch cities (the four biggest (Randstad) cities, one university city, and one industrial city): namely Amsterdam, Utrecht, Rotterdam, Den Haag, Delft, and Eindhoven. These intersections cover a wide range of crossing types and designs to ensure the generalizability of the estimation models. For each standard intersection with four arms, we can identify 12 movement directions (left/right turning or cross straight ahead per arm).

### 4.2. Delay Definition

In the conventional delay analytical approach, three delay types are distinguished, as in Figure 1. The delay event contains processes distinct from each other and has a multidimensional and nonlinear nature. The current sparse GPS data cannot capture all the details, such as when a cyclist passes the stop line of a specific arm, which can be used to compute, e.g., a stop delay and an approach delay. In this work, we creatively develop a method to define the travel time and, thus, the experienced bicycle delay to compensate for the data incompetency.

The experienced delay (*D*) is defined as the difference between the observed travel time (excluding activity travel time, see below) and the free-flow (or desired) travel time for a specific path that follows the trajectory from the upstream arrival location to the downstream departure location at the intersection (e.g., turn left/right or cross straight ahead). The central assumption is that this delay can cover all the delay components experienced by cyclists passing an intersection.

**Figure 3:**
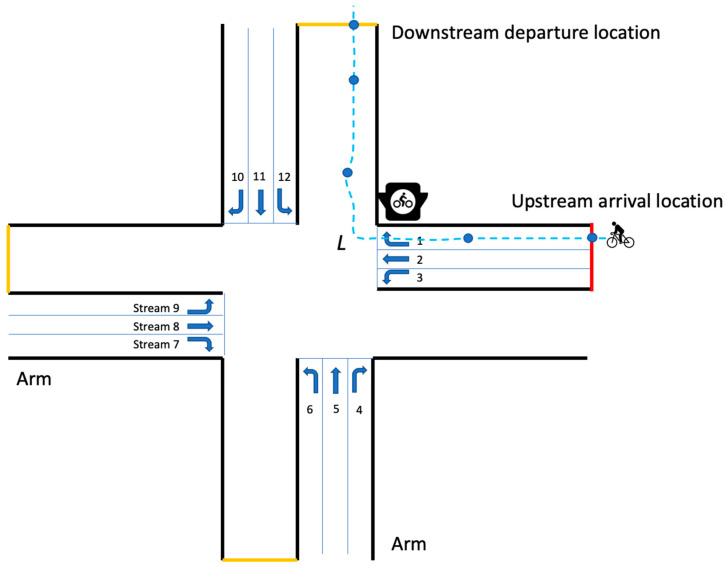
Conceptual schematic plot for bicycle flow movement at a four-armed signalized intersection (the lanes for the other modes are removed for demonstration purposes).

As shown in Figure 3, the arrival location is put at the upstream location of the incoming arm (at the upstream road segment); similarly, the departure location is set at the downstream location of the outgoing arm (at the downstream road segment). L is the cycling distance between the detectable arrival and departure locations. Note that the cycling distance for the same movement direction might vary depending on each ride’s actual reporting GPS points.

With GPS data’s longitude and latitude coordinates, we can derive the elapsed time (duration) between a detectable upstream GPS location and a detectable downstream GPS location of the intersection, considering the observed travel time (*TT*) of individual rides. We considered these travel time samples in a specific intersection to consist of two components: general cycling time and the activity duration time of this trip (e.g., cyclists might perform their errands (talking or shopping) at the intersection), inspired by that studied for cars and pedestrians [43,44]. To separate the two components, we can define a value as the maximum cycling time for a specific movement at intersections: any travel time samples larger than this value are considered to include activities during cycling. The critical step of this method is to obtain an appropriate threshold value. We can estimate this value by deriving the empirical survival functions of total travel time samples and drawing this function on semi-logarithmic paper. From the point where the curve turns into a straight line, we can assume that the distribution follows an exponential pattern. Hence, this point is an appropriate value for this threshold [43]. In this study, the value is chosen as 600 s after this process.

Note that preprocessing data operations (e.g., map-matching [20] or interpolation [5]) may be used to improve the measurement accuracy of travel times and cycling distances. This remains to be refined further in future applications. These observed travel times are considered the actual travel times experienced by cyclists for reference (as the ground truth for estimation models).

The free-flow travel time is defined as the travel distance (*L*) divided by the free-flow cycling speed (*V_free_*). So, a lower desired free-flow speed will result in a lower computed delay time, as shown in Equation (1), given the observed travel time samples (that correspond to the actual average cycling speeds on that cycle route). In this work, for demonstration purposes, we assume a uniform free-flow cycling speed of 4 m/s (obtained from the literature [45,46]) to represent the entire cycling population and to isolate the effect of desired free-flow speed heterogeneity (and also the impact of external factors on this variable).

In sum, the delay is computed as shown in Equation (1):

(1) $$D=TT-L/V_{free}$$

where *D* is the delay in s; *L* denotes the cycling distance in m; *TT* is the observed travel time (excluding activity time) between the arrival and departure locations in s; *V_free_* denotes the free-flow cycling speed in m/s. Moreover, we distinguish these travel time/delay samples according to their movement directions at intersections (numbered 1 to 12, as shown in Figure 3). Given the relatively sparse data samples, we categorize cycling movements into three main groups: right turns—R (movements 1, 4, 7, 10), through-going—T (movements 2, 5, 8, 11), and left turns—L (movements 3, 6, 9, 12), due to their specific movement features (e.g., cyclists turning right do not have to stop).

### 4.3. Influential Variables in Delay Estimation Models

In the estimation models, we have incorporated several predictors for determining bicycle delays, as highlighted in Figure 2. These include external factors such as weather conditions, temporal features, and demographic characteristics. Furthermore, intersection characteristics like layout (including the presence of tram and bus lanes) and geometric design also form a crucial part of our predictive framework. For data stratification and in-depth individual influence analysis, we have incorporated the **Intersection_ID** into our dataset.

**Weather data:** In our initial exploration of weather data as a potential determinant for bicycle delays, we considered precipitation, temperature, and wind variables. We sourced public open weather data from the Royal Netherlands Meteorological Institute (KNMI) Data Platform, downloading and combining the ASCII data files. Upon reviewing the GPS coordinates of the weather observatories, we chose to include data from several locations nearest to our specified intersections. These include Voorschoten (for intersections at The Hague), Schiphol Location 18Ct (Amsterdam), Rotterdam Location 24t (Rotterdam and Delft), Eindhoven Location A (Eindhoven), and De Bilt Location A (Utrecht). Numerous weather variables were recorded in the merged dataset, from which we selected the most representative predictors potentially related to travel delays. These include precipitation duration and intensity, temperature, average, and maximum wind speed, as further elaborated in Table 1. The weather data provided primarily represents the real-time conditions observed at the weather stations. While these readings can be useful in predicting weather conditions at nearby intersections, such interpretations must be made cautiously. This is because the physical distance between the weather station and a given intersection can introduce discrepancies in the actual weather conditions present at the intersection.

**Temporal features:** Temporal factors also play a pivotal role in our analysis, and we have included three such features: weekday number, travel hour, and a peak hour indicator.

**Demographical features:** We included the regional population as a predictor, which may impact the bicycle delay time. The assumption is that higher population levels could lead to increased congestion and, therefore, longer delay times. The population data of each region and city was sourced from the publicly accessible website (https://allecijfers.nl/, accessed on 1 July 2023).

**Intersection characteristics:** A couple of the intersection design features are considered for the ML model training, including intersection type, numbers of arms, car lanes, available bike movement streams, and the corresponding stream number based on the standard directional codes as shown in Figure 3, and dummy variables to signify the presence of tram and bus lanes.

The variables mentioned above have been summarized in Table 1.

### 4.4. Hyperparameter Tuning and Estimation Model Setup

In this study, we first separated the dataset into predictors (X) and the target variable (y). The predictors, stored in ‘X’, encompass all columns of the data except for ‘DelayTime’, which was dropped. Conversely, ‘DelayTime’ was selected as the target variable and stored in ‘y’. To divide the data into training and testing subsets, we employed a stratified sampling technique, using ‘Intersection_ID’ as the stratification criterion to ensure proportional representation of all intersections across both datasets. Consequently, each dataset reflects a balanced cross-section of intersections. The dataset is randomly split into training and testing sets for all the estimation models, with a testing size of 25%, which helps evaluate the model’s generalizability and ability to make accurate predictions on unseen data. Considering our dataset’s extensive temporal range, we anticipate that the randomly constituted training sets will encompass a comprehensive array of the characteristics present in the overall sample population.

We developed a bicycle delay estimation model, employing multi-variate linear regression as the benchmark. The model was trained using a designated training dataset, and we investigated the relevance of various features to travel delays by analyzing their respective *p*-values. The other five ML models were tuned and trained using a grid search strategy in combination with five-fold cross-validation. Cross-validation is a robust statistical technique that helps prevent model overfitting by partitioning the data into subsets, training the model on a subset, and then validating it on the remaining data (in this case—five folds are created, the method trains and evaluates the model five times, picking a different fold for evaluation every time and training on the other four folds). This approach ensures the model’s generalization ability, enhancing its predictive performance on unseen datasets, thus strengthening the reliability and credibility of the study [47]. This systematic exploration and evaluation of numerous hyperparameter combinations aimed to optimize the predictive performance of the models. The best scores achieved from the grid search, corresponding to the lowest error rates, are reported in the subsequent section. Note that all the estimation models are realized in Python, using the ‘scikit-learning’ package.

To provide an example of the hyperparameter tuning procedure, we highlight the RF model, which emerged as our project’s most effective estimation model. The grid search for this model covered several hyperparameters: `n_estimators` (the number of trees in the forest), `max_depth` (the maximum depth of each tree), `min_samples_split` (the minimum number of samples required for splitting an internal node), `min_samples_leaf` (the minimum number of samples required to be at a leaf node), and `criterion` (the metric assessing the quality of a split).

The exploration of `n_estimators` began with initial values of 50, 100, and 200. Upon noticing enhancements in model performance with the increase in this parameter, we expanded our search to include values up to 800. It was subsequently discerned that an `n_estimators` value of 800 yielded the lowest RMSE, establishing a pattern that was also found with other hyperparameters.

The best parameters identified included a `Poisson` criterion for split quality, a `max_depth` of 25 nodes, a `min_samples_leaf` of 1, a `min_samples_split` of 45, and `n_estimators` set at 800 trees. To ensure the reproducibility of results, the seed for the algorithm’s random number generator was fixed at 42. These parameters, as identified by the grid search, facilitated the highest performance from the random forest algorithm.

We extended the aforementioned optimization procedure to four other ML models. The XGBoost model, which employs an ensemble method similar to the RF model, had its hyperparameters—`n_estimators`, `max_depth`, `min_samples_split`, `min_samples_leaf`, and `learning_rate`—tuned. The optimal hyperparameters identified were a learning rate of 0.1, a maximum tree depth of five nodes, a minimum of four samples required at a leaf node, a minimum of two samples necessary for splitting an internal node, and 50 trees in the boosting process.

For the kNN model, we tuned the number of neighbors used for the majority voting process (`n_neighbors`), the type of distance metric used (`p`), and the function used for weighting the influence of neighbors (`weights`). The optimal parameters determined were nine neighbors, a Minkowski distance parameter ‘p’ set to one (which equates to using the Manhattan distance), and uniform weights, signifying all points in each neighborhood are equally weighted.

In tuning the SVR model, we focused on the hyperparameters `C` (the penalty for misclassification), `gamma` (the influence of individual training examples on the decision boundary), `epsilon` (the degree of insensitivity to errors), and `kernel` (the kernel function). The optimal parameters for the SVR model were identified as a `C` value of 0.1, an `epsilon` of 0.3, a `gamma` of ‘scale’, and a `kernel` of ‘rbf’.

Lastly, for the NN model, we sought an optimal configuration consisting of the number and size of hidden layers (`hidden_layer_sizes`), activation function (`activation`), the solver for weight optimization (`solver`), learning rate, and an L2 penalty (regularization term) `alpha` via a grid search. The optimal setup with the highest performance consisted of two hidden layers with 50 and 100 neurons, respectively, ‘tanh’ as the activation function, ‘sgd’ as the solver, a constant learning rate, and an L2 penalty alpha of 0.05.

### 4.5. Performance Indicators

In the evaluation of the estimation models, two key performance indicators, R-squared (R^2^) and root mean square error (RMSE), are employed to assess the model’s predictive accuracy and reliability for both training and testing sets. The R-squared metric, also known as the coefficient of determination, quantifies the proportion of the variance in the dependent variable (bicycle delay) that is predictable from the independent variables. This metric measures how well-observed outcomes are replicated by the model based on the proportion of total variation in outcomes explained by the model. A higher R^2^ indicates that the model’s estimation aligns more closely with the observed data, signifying a more accurate model.

RMSE, on the other hand, measures the average magnitude of the prediction error, i.e., the differences between the predicted and observed values. It quantifies the model’s predictive error, which is directly linked to the concept of reliability. A smaller RMSE indicates a model that reliably produces less error, implying better reliability. Furthermore, because RMSE penalizes larger errors more severely, models that minimize RMSE are especially desirable when large prediction errors are particularly problematic. In contrast, metrics like MAE (mean absolute error) would provide less significant penalization for larger errors; MSE (mean square error) does not provide the intuitive scale as RMSE does; MAPE (mean absolute percentage error) can heavily penalize small deviations when the actual values are low, which is not ideal for our application. Therefore, they are not selected as error indicators. This study applied a log transformation (the natural logarithm of one plus the input array) to improve the model’s performance to the delay outcome variable. This transformation serves to temper the influence of extremely high values, resulting in a more symmetrical distribution and, thus, making it more amenable to modeling [48]. However, due to this transformation, interpreting the R-squared and RMSE values should be cautiously approached as they now represent relationships in the log scale [48].

**Figure 4:**
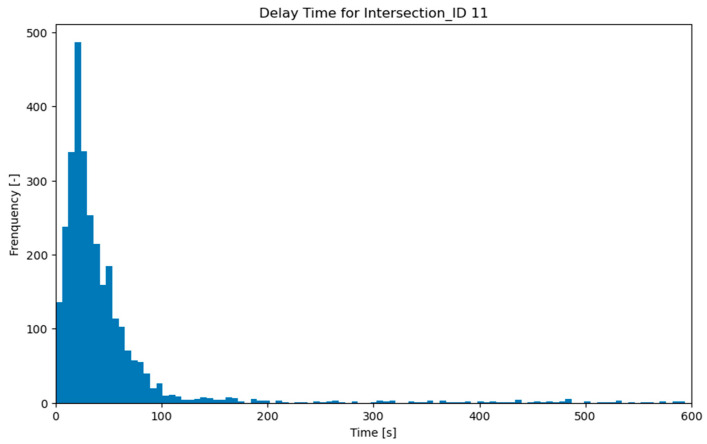
Distribution of the frequency of delay time at intersection ID 11.

A comparative analysis of the distribution of the predicted and original delays was conducted using the testing set to assess the model performance further. This involved visualizing the distributions of both data sets to observe any differences or similarities. Given the significant right-skewed distribution of the data (reflecting a Poisson queuing/waiting process; see Figure 4), the median was chosen as a more robust measure of central tendency compared to the mean. Therefore, the medians and their RMSE values of the predicted and original delays were compared to provide a more accurate representation of the central location of the data. This approach helps to mitigate the influence of outliers and provides a more reliable comparison between the predicted and actual delay values.

## 5. Results and Discussion

This section starts with an exploratory analysis of the concerned data, and it is followed by the training results, with a zoom into the best-performing model among the developed models and its estimation implications. This section ends with a reflection on insights relevant to bicycle delay estimation.

### 5.1. Exploratory Data Analysis

The exploratory dataset contains 21,111 travel record entries. We identified missing values among the weather predictors; they accounted for a negligible portion of the total data, less than 1.14%. A decision had been made to exclude these records, aiming at a more accurate estimation. Upon applying a travel time threshold of 600 s, the total number of travel records was reduced to 19,406. The largest portion of the travel records was attributed to Intersection 11 (one of the busiest intersections in Amsterdam: the Ferdinand Bolstraat-Stadhouderskade), with 3488 entries accounting for 17.8% of the total. Conversely, Intersection 6 (in Delft) had the smallest data share due to a lower penetration, with 168 entries. The mean value of the delay was found to be 70.3 s, while the median was 34.0 s. Before implementing the data training approaches, we visualized the outcome variable: delay time. Each intersection consistently exhibited a right-skewed distribution, closely resembling a Poisson distribution (an example of Intersection 11 is given in Figure 4). Additionally, an overview of the layout and geometrical characteristics as well as the related data sample size from each intersection is in given in Table 2.

Our initial exploratory analysis employed a multivariate LR model, aiming to predict bicycle delay time. This benchmark model offered an overall R^2^ value of 0.040 and RMSE of 1.126, suggesting the model’s modest performance.

An examination of the *p*-values revealed that the majority of the predictors demonstrated statistical significance (*p* < 0.05). This set of significant predictors included weather conditions (specifically ‘Precipitation_Intensity’ and ‘Temperature’), temporal features (‘Weekday_Number’, ‘Hour’, and ‘Peak’), and other variables such as ‘Intersection_ID’, ‘Intersection_Type’, ‘Population’, ‘Car_Lanes’, ‘Bike_Streams’, ‘Tram_Dummy’, and ‘Bus_Dummy’. However, the significance of variables like ‘Precipitation_Duration’ (*p*-value = 0.150), ‘Wind_Average_Speed’ (*p*-value = 0.362), ‘Wind_Maximum_Speed’ (*p*-value = 0.656), and ‘Stream_Number’ (*p*-value = 0.836) was weaker, though still considered relevant to bicycle delay estimation (in particular, the contribution of ‘Stream_Number’ might not be captured in the LR model). ‘Arms’, with a *p*-value of 0.885, was the least significant among the considered variables. These variables’ interaction with delay estimation will be further explored during the ML modeling process.

A closer inspection of the coefficients revealed interesting patterns: ‘Stream_Number’, ‘Weekday_Number’, ‘Precipitation_Intensity’, ‘Wind_Maximum_Speed’, and ‘Arms’ displayed negative coefficients, suggesting an inverse relationship with bicycle delays. The other predictors showed positive coefficients, implying that an increase in these variables likely correlates with a delay increase.

This exploratory analysis served as our benchmark before proceeding to the more complex ML models. The objective was to understand the data better, identify potential key predictors, and develop a sense of the delay’s underlying dynamics. The significant variables found in this initial model were expected to bear relevance in our subsequent ML analyses.

### 5.2. ML Model Training and Testing Results

Upon fitting all the concerned models to the data, we compared their accuracy using R^2^ and RMSE between training and testing sets. We drew insights from these comparisons, considering each model’s complexity. The metrics for all the models are outlined in Table 3.

The RF model appears to be the most effective, with the highest R^2^ value of 0.347 and the lowest RMSE, indicating that approximately 34.7% of the variance in the dependent variable is predictable from the independent variables. The RMSE of the RF model on the training data is 0.944 (log), which is the lowest among all models, implying the best performance in terms of prediction accuracy. This is closely followed by the XGBoost model. Other models, including linear regression, kNN, and NN, demonstrated similar performance, albeit with lower R^2^ and higher RMSE scores. However, the SVR model stands out with the highest error rate. The performance drops from the training set to the testing set for all models, but still with the RF model as the top performer.

Based on these performance metrics, we have selected the RF model as our best model for further analysis. We will also investigate its feature importance to understand the factors contributing most to bicycle delays.

### 5.3. Best Performance Model and Its Implications

In the training results, it is worth noting that the R^2^ values are relatively low. The R^2^ statistic, also known as the coefficient of determination, measures how well the variations in the outcome variable (in this case, bicycle delay time) are explained by the model’s predictors.

The best-performing model, random forest, has an R^2^ score of 0.097, which suggests that the model explains approximately 10% of the variation in bicycle delays. Although this might seem low, it is crucial to consider the complexity of the problem we are addressing. Predicting bicycle delays depends on numerous intricate factors, including weather, time of day, demographic characteristics, and intersection properties. Creating a model that can effectively account for all these factors is often challenging.

The remaining 90% of unexplained variation could be attributed to factors not included in our models, such as local traffic conditions, controller signals, pavement material and visibility, unmeasured environmental factors, individual bicyclist behavior, or inherent randomness in the process due to the absence of these data sources. Low R^2^ scores are common in real-world prediction tasks, mainly when dealing with complex systems with many underlying influencing factors, as shown in Figure 2.

**Figure 5:**
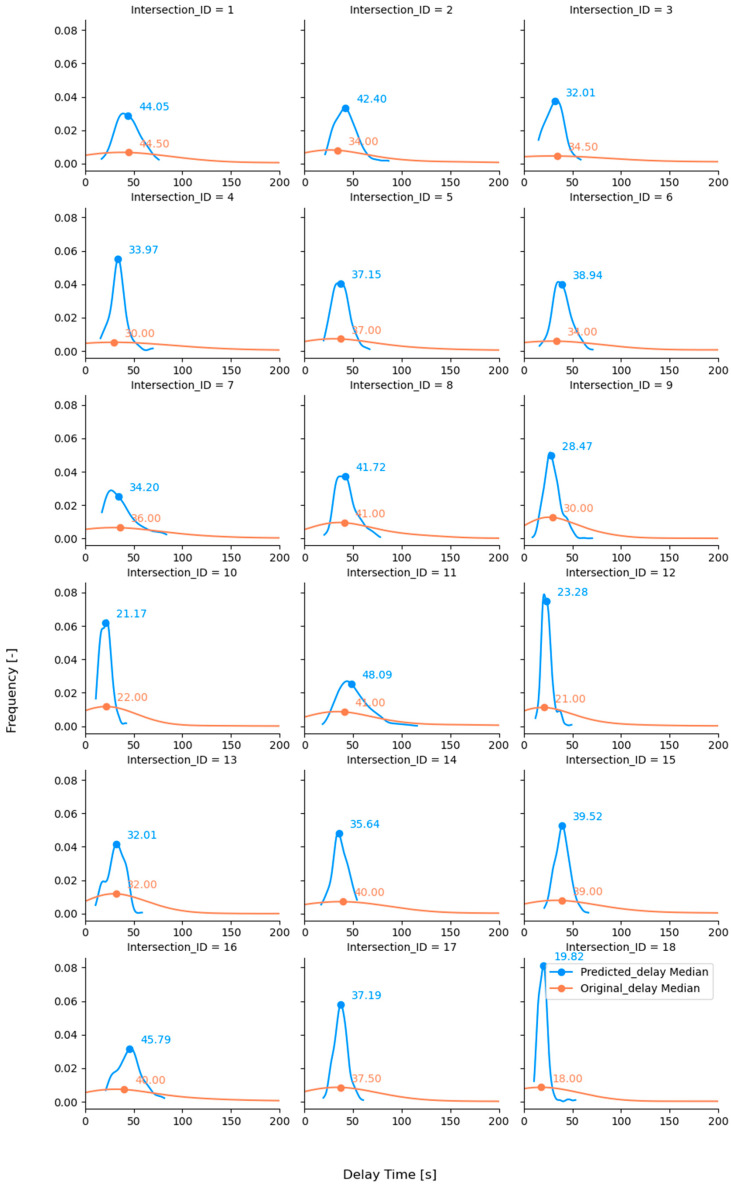
Distribution of the predicted (in blue) and original (in red) delay time per intersection (unit in the horizontal axes: second).

To delve deeper into the results from the RF model, we visualized the density distribution of the actual and predicted results in the testing sets, with the medians stratified by intersection ID (see Figure 5). From this data visualization, it is evident that the RF model training aims to generate predictions that closely align with the actual real-world bicycle delays. The RF model could closely capture the median delay time at most intersections. However, there might be a loss of variance, suggesting that our model may not capture the full range of delay times as disparity exists (to a certain extent) between the individual predicted and original delays. Note that the magnitude of bicycle delays derived from this ML model (the median values of bicycle delays at the 18 intersections range from 19.82 s to 48.09 s, with standard deviations of a range between 5.54 s and 16 s) is of a similar order as the result reported in a previous empirical study [5] (the median values of bicycle delays at the concerned intersections range from 16 s to 57 s, with standard deviations of a range between 15 and 36 s). Moreover, according to the sample stream number, the model can distinguish three cycling movements (right turns, left turns, and through-going). Thus, the delay distribution per movement can be generated to reflect specific turn maneuvers.

Interestingly, the *t*-test revealed no significant difference between the observed and predicted results (with a *p*-value of 0.355). This statistical test supports the strength of the RF model, demonstrating its ability to predict median delay times that are statistically indistinguishable from the actual observed medians. The RMSE of the delay medians from all the intersections is less than 4 s (3.62 s). This suggests that our model, while perhaps not capturing the total variability in delay times, can reliably predict the ‘typical’ delay overall, regarding three distinct movement directions at different intersections. This is valuable information for policymakers and transport authorities in understanding and managing average delays at these junctions.

**Figure 6:**
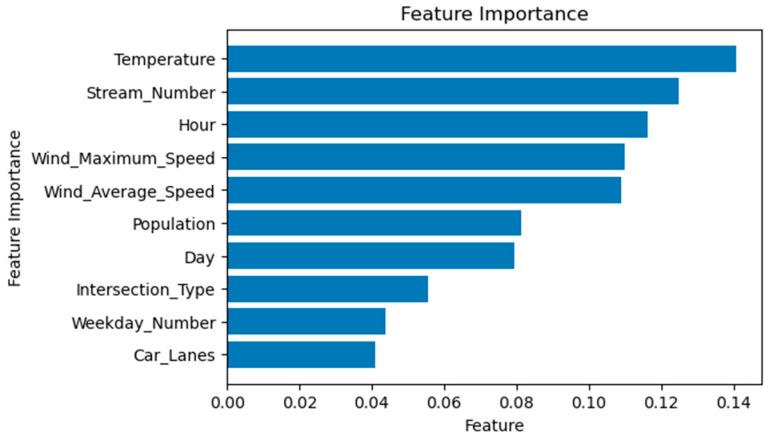
Feature importance (random forest algorithm).

To further explore the influence of different features on bicycle delays, we derive the feature importance from the ‘sklearn’ package. Figure 6 shows that the temperature variable (‘Temperature’) is the most significant predictor in the RF model. The ‘Stream_Number’ ranks second and can well reflect the turning typology. The wind variables (‘Wind_Maximum_Speed’ and ‘Wind_Average_Speed’) also show their significance. Additionally, temporal features such as Hour, Day, and Weekday_Number have substantially impacted the model training process. This suggests that certain weather conditions, specifically temperature and wind speed, play a major role in determining bicycle delays. The time of travel, be it the hour of the day or day of the week, also significantly impacts delay times.

**Figure 7:**
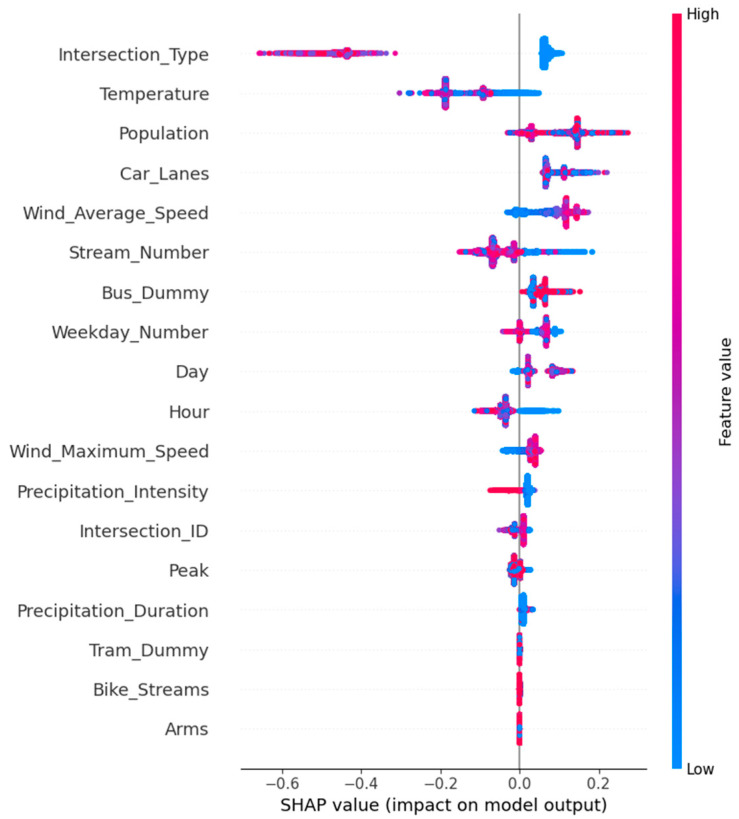
SHAP summary plot of feature impact.

To better understand the predictors’ impact on bicycle delays, we utilized the SHAP (SHapley Additive exPlanations) package. This tool provides a unified measure of feature importance and allows for the interpretation of the contribution of each feature to the prediction for each sample. We visualize each feature’s impact on the model’s prediction in Figure 7. The *x*-axis indicates the magnitude of influence, and the color indicates the feature value. Features are ordered from top to bottom by their overall importance level, and the placement of dots on either side of the center reflects whether a feature value increases or decreases the prediction.

**Factors of increased delays**: From the SHAP output, we identified several features that may increase the delay time. These include low temperatures, morning hours (as indicated by low values), wind speed, and high precipitation intensity. These factors may contribute to slower cycling speeds or increased congestion, thereby increasing delay times.

Low temperatures (e.g., lower than 11 °C—the Dutch average annual temperature) may increase delay times due to various factors. Cold weather can make cycling more challenging and less comfortable, potentially slowing down cyclists. Additionally, certain weather conditions associated with low temperatures, such as ice or snow, can create hazardous road conditions that require cyclists to slow down for safety.

Morning hours, particularly during the rush hour (between 8:00 and 8:30 [2]), are typically associated with higher traffic volumes as people commute to work or school. This increased congestion can lead to slower cycling speeds and longer delay times. Moreover, cyclists may also need to exercise more caution during these busy hours, further contributing to delays.

Wind can also increase delay times, mainly if it is a headwind. Cycling against the wind requires more effort and can significantly slow down a cyclist’s speed. Even a moderate wind can make a noticeable difference in travel times.

Precipitation could be another factor in bicycle delays. The conditions of heavy rainfall typically correlate with extended bicycle travel times. This could be due to several factors, such as the need to navigate more cautiously, changes in traffic light patterns to accommodate different traffic conditions, or increased vehicle congestion due to unfavorable weather conditions.

Moreover, Mondays might be associated with increased delay times for several reasons. After the weekend, there could be an increased volume of commuters, leading to more traffic congestion. Additionally, logistical activities such as deliveries or construction are often scheduled for the start of the week could contribute to delays. Fridays might contribute to decreased delay times because most people might leave work early or have a modified schedule on Friday.

**Factors of decreased delays**: Conversely, certain features were potentially found to decrease the delay time. These include moderate and high temperatures and off-peak hours. These conditions may lead to faster cycling speeds or reduced congestion, resulting in shorter delays.

To capture the variations in bicycle delays, it is important to consider specific turn maneuvers and turning typology within the junction. Different types of turns (e.g., left turns, right turns, and crossings) are deemed to have distinct delay characteristics due to variations in signal timings and conflicts with other modes of transportation. Particularly, cyclists turning right usually do not have to stop and thus experience minimal delays, whereas cyclists turning left or going straight may have to wait for a green light. This characteristic has been well captured by the feature ‘Stream_Number’. However, this observation should be interpreted with caution since this variable was found to be the weakly significant variable in the LR model.

Interestingly, several features appeared to have little or no relationship with the delay time. These include the presence of bus lanes, the presence of trams, and the number of arms at intersections. The lack of a significant relationship suggests that these factors may not substantially impact bicycle delays in the context of our model.

This is valuable information for cyclists, traffic planners, and policymakers alike. For instance, knowing that higher winds or precipitation traveling times are associated with increased delays can inform decisions about infrastructure improvements or traffic management strategies. Understanding the role of temporal variables (peak/off-peak, weekdays) and turning typology can help plan for expected delays during certain times and inform strategies to manage and reduce such delays.

### 5.4. Reflection on Relevant Insights

In this case study, we successfully demonstrate how cycling data can provide insights for traffic management and policymaking, using an innovative ML-based case study on bicycle delay estimation, amongst many possible use cases within the bicycle data-driven application framework. While we do not aim to develop the most accurate estimation model for monitoring bicycle delay per movement direction at signalized intersections for operational purposes (e.g., minimizing prediction errors for individual delay samples), we show that one of the ML models, the RF model, can estimate and predict the average magnitude (regarding the mean, median, and distribution) of this crucial variable to a high accuracy level (R^2^ score of 10%, relatively low validation error—less than 4 s regarding the RMSE of delay medians). Also, the predicted delay magnitude falls within the reported value range from a previous empirical study [5]. The significant features (e.g., in Figure 6) of the delays identified in the ML models are consistent with the significant, influential variables specified in the simple linear regression model. This method only uses sparse cycling GPS data combined with publicly accessible information, such as weather information, and intersection complexity, leveraging the burden of understanding local conditions (traffic signals, flow demand, saturation level).

The application and results of our estimation models can offer data-driven insights and evidence-based information for policymakers and cooperating authorities. It enables them to understand average bicycle delays and their distributions at specific intersections or even on a national level.

In the Netherlands, intersections experience high bicycle demand, sometimes surpassing the number of cars served. This suggests a potential paradigm shift where intersection efficiency is measured not just by car volume but by the total number of people served across all transportation modes. To assess the overall time lost at intersections, understanding bicycle delays is crucial. With this knowledge, prioritizing bicycle traffic becomes a possibility through strategies like allocating more green time or implementing dedicated cycle paths. However, such adaptations may impact other traffic users, potentially causing delays for cars. Policymakers must weigh these considerations against equity concerns for all road users and align decisions with specific policy objectives and desired service quality. Our contribution to this decision-making process lies in unraveling information on bicycle delays.

Notably, the issue of bicycle delays at intersections is somewhat acknowledged by formulating practical guidelines, for example, in the Netherlands, where several municipalities define critical values for the evaluation of waiting times for bicycle traffic at signalized intersections (in the city of Arnhem, for a bicycle-friendly intersection, the average waiting time should be less than 15 s; a maximal bicycle waiting time of 60 s is strived for in the city of Maastricht, while in Tilburg, this maximum value varies between 35 s and 80 s) [49]. Such knowledge, derived from our proposed estimation models, is crucial when establishing priority and function maps for bicycle users (and car users too). It provides a comprehensive picture of where and when delays are most likely to occur. This can aid authorities in developing and implementing strategic traffic management measures and guidelines tailored for specific and generic intersections.

By leveraging ML’s ability to analyze and predict complex patterns from available traffic data sources, authorities and policymakers can create more efficient, sustainable, and user-friendly transport systems. The potential improvements in traffic management drawn from this study could enhance the commuting experience for thousands of individuals, reduce congestion, promote cycling as a sustainable mode of transportation, and contribute towards a greener urban environment. In this way, ML modeling not only helps us understand our present transportation challenges but also enables us to create solutions for a smoother, more efficient future.

## 6. Conclusions and Outlook

This article addresses the need for a generic framework to harness cycling data and extract traffic management and policy-relevant insights. Specifically, it focuses on the application of estimating average bicycle delays at signalized intersections. The study proposes ML-based approaches using GPS cycling data from the Talking Bikes program. Amongst various ML models, the random forest model outperforms the others due to a higher accuracy level. Insight revealed from this estimation model, such as that traveling with higher winds or precipitation is associated with increased delays and moderate temperature or off-peak hours are connected with decreased delays, can inform decisions about infrastructure improvements or traffic management strategies. Understanding the role of temporal/weather variables and turning typology can help in planning for expected/predicted delays during certain times/conditions and can inform strategies to manage and reduce such delays at specific intersections or on a national level.

The study demonstrates the feasibility of estimating bicycle delays using only sparse GPS cycling data and publicly accessible information, and it emphasizes the potential of data-driven approaches in informing traffic management, bicycle policy, and infrastructure development. The estimation models for bicycle delays at signalized intersections can be enhanced in the following manners and provide more accurate, operational- and policy-relevant results.

**Refined definition of delays:** Currently, the delay time is defined based on a reference desired (free-flow) cycling speed. It is recommended to derive this value from the empirical dataset to enhance the accuracy of delay samples.

**Expansion of intersection coverage:** To enhance the generalizability of the estimation models, it is recommended to include a larger number of intersections and cities. By covering a broader range of intersection types and contexts, the models can better account for the diversity of cycling conditions and traffic dynamics. This expansion will contribute to more robust and reliable estimates of bicycle delays at signalized intersections.

**Inclusion of local control signals and traffic count information:** Local control signals provide detailed information about signal timings and phase sequences specific to each intersection. Incorporating this data will enhance the accuracy of delay estimations. Count information, including the number of bicycles and other road users passing through the intersection, will further refine the estimation models and improve their predictive capabilities.

In addition to specific improvements to enhance the capabilities of the ML models to estimate bicycle delays, similar models can be applied to capture bicycle delays, travel times, and demand on bicycle paths and highways. Filtering the data slightly differently would allow us to reformulate this algorithm specifically for bicycle corridor delays.

Moreover, enhancements in the information quality provided by the underlying sensor system, including aspects such as accuracy, reliability, sampling rate, aggregation level, latency, and privacy, could substantially improve the efficacy of our analyses and estimation tools. Investigating the potential integration of advanced sensor technologies or protocols within bicycle network [4,22,50] may yield richer and more granular data, thereby enhancing the overall reliability and precision of insights derived from the sensor system.

These improvements to the delay estimation algorithms and the underlying sensor network will contribute to better-informed decision-making processes for multi-modal area-oriented utilization, allowing for optimized traffic signal timings, improved infrastructure design, and enhanced overall cycling experience at intersections.

## Figures and Tables

**Table 1:** Overview of influential variables used in the machine learning model.

	Predictors	Data Type	Details	Sample Record
Intersection identifier	Intersection_ID	Integer	Intersection index	1
Weather conditions	Precipitation_Duration	Integer	Duration of precipitation in s over 10 min	600
	Precipitation_Intensity	Decimal	Precipitation intensity over 10 min (mm/h)	2.41
	Temperature	Decimal	Average air temperature in °C over 10 min	13.30
	Wind_Average_Speed	Decimal	Average wind speed in m/s over 10 min	8.47
	Wind_Maximum_Speed	Decimal	Max. actual wind speed in m/s over 10 min	12.43
Temporal features	Weekday_Number	Integer	Day of the week of the travel record (with 1 denoting Sunday, 2 denoting Monday, and so forth until 7 [Saturday])	1
	Hour	Integer	Hour of the day of the travel record	13
	Peak_Dummy	Dummy	Peak hour indicator (1: peak for the period between 7:00 and 19:00; 0: otherwise)	1
Demographic feature	Population	Integer	Population of the region/city	651,157 (Rotterdam in 2020)
Intersection characteristics	Intersection_Type	Integer	Intersection type (Four-armed: 1, three-armed: 2, or roundabout: 3)	1
	Stream_No.	Integer	Standard index of bike flow movements at intersections	2
	Arms	Integer	Total No. of arms	4
	Car_Lanes	Integer	Total No. of car lanes	15
	Bike_Streams	Integer	Total No. of bike streams	12
	Tram_Dummy	Dummy	The presence of a tram line (1: presence)	1
	Bus_Dummy	Dummy	The presence of a bus lane (1: presence)	0

**Table 2:** Intersection characteristics and the related data sample size. Note, # denotes No.

Int. ID	City	Int. Type	#Arms	#CarLanes	#Bike Streams	Tram Dummy	Bus Dummy	#Trip Records
1	Rotterdam	1	4	15	12	1	0	542
2	Rotterdam	3	4	14	8	1	1	498
3	Rotterdam	1	4	4	12	1	1	898
4	Delft	3	4	12	8	1	1	225
5	Delft	1	4	12	12	1	1	253
6	Delft	2	3	4	6	0	0	168
7	The Hague	1	4	9	12	1	1	899
8	The Hague	1	4	6	12	1	1	588
9	The Hague	2	3	8	6	1	0	637
10	Amsterdam	1	4	5	12	1	1	3007
11	Amsterdam	1	4	8	12	1	0	3488
12	Amsterdam	2	3	6	6	1	0	2420
13	Eindhoven	1	4	4	12	0	1	995
14	Eindhoven	1	4	12	12	0	1	755
15	Eindhoven	1	4	13	12	0	1	242
16	Utrecht	1	4	4	12	1	1	1362
17	Utrecht	1	4	15	12	0	1	1650
18	Utrecht	2	3	6	6	0	0	779

**Table 3:** Training and testing performances of estimation models.

	Training		Testing		
Estimation Models	R^2^	RMSE (log)	R^2^	RMSE (log)	RMSE (Median)
Linear regression	0.045	1.141	0.040	1.126	
Random forest (RF)	0.347	0.944	0.097	1.092	3.62 (s)
Gradient boosting trees (XGBoost)	0.211	1.038	0.091	1.096	
Support vector regression (SVR)	0.007	1.146	0.007	1.164	
K-nearest neighbors (kNN)	0.196	1.048	0.040	1.157	
Neural networks (NN)	0.095	1.111	0.060	1.115	

## Data Availability

Data are contained within the article.
